# Antimicrobial Resistance Profile and Biofilm Production of Microorganisms Isolated from Oropharynx of *Rupornis magnirostris* (Gmelin, 1788) and *Caracara plancus* (Miller, 1777)

**DOI:** 10.1155/2020/8888618

**Published:** 2020-07-10

**Authors:** Fernanda Alda da Silva, Sandrelli Meridiana de Fátima Ramos dos Santos Medeiros, Sérgio Dias da Costa-Junior, Ana Emília Medeiros Roberto, Sarah Brandão Palácio, Reginaldo Gonçalves de Lima-Neto, Rejane Pereira Neves, Carolina Peixoto Magalhães, José Eduardo Garcia, Isabella Macário Ferro Cavalcanti

**Affiliations:** ^1^Universidade Federal de Pernambuco (UFPE), Laboratório de Microbiologia e Imunologia, R. Alto do Reservatório—Alto José Leal, SN, Vitória de Santo Antão, PE 55608-250, Brazil; ^2^Universidade Federal de Pernambuco (UFPE), Laboratório de Anatomia, R. Alto do Reservatório—Alto José Leal, s/n, Vitória de Santo Antão, PE 55608-250, Brazil; ^3^Universidade Federal de Pernambuco (UFPE), Setor de Microbiologia, Laboratório de Imunopatologia Keizo Asami (LIKA), Av. Moraes Rego, 1235, Cidade Universitária, Recife, PE 50670-901, Brazil; ^4^Universidade Federal de Pernambuco (UFPE), Laboratório de Micologia Médica do Departamento de Micologia, Av. Prof. Moraes Rego, s/n, Cidade Universitária, Recife, PE 50760-420, Brazil; ^5^Universidade Federal de Pernambuco (UFPE), Laboratório de Culturas e Pesquisas in vitro, Departamento de Medicina Tropical, Av. da Engenharia, s/n, Cidade Universitária, Recife, PE 50670-420, Brazil; ^6^Universidade Federal de Pernambuco (UFPE), Laboratório de Biotecnologia e Fármacos, R. Alto do Reservatório—Alto José Leal, SN, Vitória de Santo Antão, PE 55608-250, Brazil

## Abstract

The aim of this preliminary study was to identify microorganisms with antimicrobial resistance profile and biofilm producers in oropharynx of *Rupornis magnirostris* and *Caracara plancus*. Six *R. magnirostris* and six *C. plancus* maintained in Triage Center for Wild Animals (CETAS) facilities were studied. Coagulase-positive staphylococci (CoPS), enterobacteria, and yeasts were identified by the biochemical analysis or MALDI-TOF mass spectrometry. The resistance profile of the microorganisms was analyzed according to CLSI. The biofilm production was evaluated by Congo red and violet crystal staining methods. Among the 12 birds, 10 presented strains of CoPS and/or enterobacteria with resistance profile, such as methicillin-resistant CoPS (MR-CoPS), vancomycin-resistant CoPS (VR-CoPS), extended-spectrum *β*-lactamase-producing *Enterobacteriaceae* (ESBL), and *Klebsiella pneumoniae* carbapenemase- (KPC-) producing bacteria. Regards the fungal analysis, *Candida* spp., *Cryptococcus* spp., *Rhodotorula mucilaginosa*, *R. glutinis*, and *Trichosporon coremiiforme* were identified. All the *Trichosporon coremiiforme* strains were resistant to amphotericin B, as well as all the *Rhodotorula mucilaginosa* exhibited resistance to fluconazole. Related to the biofilm production, among the 8 CoPS, 27 enterobacteria, and 10 yeasts isolates, 3, 16, and 7 strains were biofilm producers, respectively. Thus, the presence of these microorganisms in birds of prey is worrisome, highlighting its possible influence in the spread of infections in urban centers.

## 1. Introduction

The hawk-carijó (*Rupornis magnirostris*) and the carcará (*Caracara plancus*) belong to the orders Falconiformes and Accipitriformes, respectively. Both species are widely distributed throughout the Americas, mainly in South America, and are well distributed in the Brazilian territory [1]. These animals inhabit different sorts of environments including urban areas, as well as hang around roadsides, forest edges, and pastures in search of food. These birds of prey have an important ecological role in the control of the numbers of the small animals, being considered top predators of food chains and webs, helping to maintain stable the ecological balance of the ecosystem, in which they live [2].

Birds of prey are considered bioindicators of environmental quality, due to their ecological role, but they are also affected by changes in the environmental conditions and can be indicators of the preservation of the habitat, to which they are inserted [2, 3]. In addition, some species, such as *Rupornis magnirostris* and *Caracara plancus*, can adapt to anthropogenic environments, thus being exposed to a wide assortment of chemical and biological compounds present in this environment [4]. Free-living birds of prey, as well as other groups of birds, can be considered reservoirs or even vectors of pathogens of importance for the poultry farms, since serological studies show that they are exposed to micro-organisms by contact with residues and farm runoff, by ingestion of contaminated carcasses, or by the contamination of heavy metals dispensed in the environment [3, 5].

This contact with anthropic areas, feeding on remnants of possibly contaminated human waste, also increases the contact of these birds of prey with antimicrobial resistant microorganisms [6–9]. In 2012, Radhouani et al. [6] identified multiresistant *E. coli* and enterococci isolates from faecal samples of the common buzzards (*Buteo buteo*), a medium to large bird of prey. Sousa et al. [7] suggested that birds of prey, such as *Buteo buteo*, *Strix aluco,* and *Corvus corone*, seem to be a reservoir of multidrug-resistant *S. aureus* and coagulase-negative staphylococci. Our research group has identified *Enterobacteriaceae* with resistance profile in rectal swabs present in gavião-carijó (*Rupornis magnirostris*), among them, *E. coli*, *K. pneumoniae*, and *Salmonella* spp. are resistant to ciprofloxacin and lots of beta-lactams [8]. These animals, besides being affected by infections caused by these microorganisms, can become reservoirs and potential disseminators of these agents owing to their migratory capacity [6–9].

Besides the possible colonization of these birds by resistant micro-organisms, these infectious agents may also be producers of virulence factors, such as biofilm. A biofilm consists of a group of sessile cells, mono- or multispecies, and may be of bacterial or fungal origin, which are adhered to a biotic or abiotic surface and surrounded by an organic polymer matrix, the exopolysaccharide (EPS). The physical structure of the biofilm contributes to antimicrobial resistance in some species of microorganisms and may explain the chronicity or recurrence of infections since microorganisms in a biofilm tend to be more resistant to antimicrobial agents and therefore require higher concentrations of these agents to inhibit or eradicate them [10].

Since the identification of microorganisms resistant to antimicrobials and biofilm producers in birds of prey is scarce, this preliminary study aims to identify these pathogens isolated from the oropharynx of *Rupornis magnirostris* and *Caracara plancus*. In this way, we intend to fill this gap in the scientific field and contribute to public health, since these species are present in anthropic environments and can become disseminators of these pathogens.

## 2. Materials and Methods

### 2.1. Animal Obtention

Six *Rupornis magnirostris* and six *Caracara plancus* were used, with different weights and ages, obtained from the Wild Animals Triage Center of the Environment agency of the Pernambuco (CETAS)–IBAMA (Brazilian Institute of the Environment and Renewable Natural Resources). The animals donated by the center are not able to be reinserted in nature due to mutilations in the wings or legs that prevent their free locomotion. These animals were assigned by the CETAS to the collection of the samples one day after they had been rescued from the nature, and they had not undergone antibiotic therapy. The research project has the license granted by the Ethics Committee on the Use of Animals (CEUA), the Center for Biosciences (CB) of UFPE, receiving the appropriate authorization in the process number 23076.045832/2016-42 and authorization of SISBIO with number 57230-1.

### 2.2. Samples Collection

The oropharynx samples were collected using sterile swabs, which were transferred to brain heart infusion broth (BHIB) (HiMedia®, Mumbai, India) for the identification of coagulase-positive staphylococci (CoPS), tetrathionate broth (HiMedia®, Mumbai, India) for the identification of *Enterobacteriaceae*, and Sabouraud broth (HiMedia®, Mumbai, India) for the identification of yeasts. All samples were transported in a polystyrene box, suitably sealed to the Laboratory of Microbiology and Immunology of the Centro Acadêmico of Vitória, at the Federal University of Pernambuco (CAV/UFPE), and incubated at 35 ± 2°C for 24 h for later identification of the microorganisms.

### 2.3. Identification of Coagulase-Positive Staphylococci (CoPS)

Samples from the BHIB were seeded on Baird Parker agar (HiMedia®, Mumbai, India) at 35 ± 2°C for 48 h for identification of CoPS. After incubation, the typical colonies of CoPS were submitted to Gram staining and biochemical tests such as catalase, coagulase, DNase, and mannitol production to confirm the presence of CoPS [11].

### 2.4. Identification of *Enterobacteriaceae*

Samples from tetrathionate broth were plated on MacConkey (Kasvi®, Paraná, Brazil) agar and *Salmonella Shigella* agar (SS) (Kasvi®, Paraná, Brazil) and reincubated at 35 ± 2°C for 24 h. After incubation, the samples that presented microbial growth compatible with *Enterobacteriaceae* were submitted to biochemical tests, such as oxidase, lysine, urea, citrate, triple sugar iron (TSI), motility-indole-ornithine (MIO), phenylalanine, DNase, sucrose, and arabinose [12].

### 2.5. Identification of Yeasts

Homogenous inocula of yeast cells were grown and maintained on yeast extract peptone dextrose agar medium (YEPD) (HiMedia®, Mumbai, India). Incubations were standardized at 24 h, and strains were grown aerobically at 35 ± 2°C. In order to avoid changes in the protein expression pattern, the culture conditions and growth time were standardized as described above. All cultures were checked for purity prior to mass spectrometry (MALDI-TOF Autoflex III Bruker Laser Nd: YAG smartbeam, Bruker Daltonics Inc., USA/Germany) [13].

One single colony was directly deposited onto a 196 position target plate (Bruker Daltonics GmbH), and two such deposits were made for each isolate. Aliquots of 1 *μ*L of 70% formic acid were added and mixed gently with the yeasts. When the liquid medium was almost evaporated, the preparation was overlaid with 1 *μ*L of saturated matrix solution (75 mg/mL of *α*-cyano-4-hydroxycinnamic acid (CHCA) in ethanol/water/acetonitrile (1 : 1 : 1) with 0.03% trifluoroacetic acid (TFA) (Merck®, New York, USA). A total of 20 isolates (2 × 20 spots) were deposited per plate, and the matrix-sample was crystallized by air-drying at room temperature for 5 minutes [13].

The equipment used was MALDI TOF Autoflex III mass spectrometer (Bruker Daltonics Inc., USA/Germany) equipped with a Nd: YAG (neodymium-doped yttrium aluminium garnet; Nd : Y_3_Al_5_O_12_) laser of 1064 nm, set to a 66% power. The mass range from 2,000 to 20,000 Da was recorded using a linear mode with a delay of 104 ns and an acceleration voltage of +20 kV. The resulting peak lists were exported to the software MALDI Biotyper™ 3.0 (Bruker Daltonics, Bremen, Germany) where the final identifications were achieved.

### 2.6. Identification of Resistance Profile

#### 2.6.1. Antimicrobial Susceptibility Tests for CoPS

The identification of resistance profiles of the micro-organisms was performed according to the Clinical and Laboratory Standards Institute [14]. Samples positive for CoPS were submitted to the disc diffusion method, as well as the microdilution test and agar screening, for the identification of methicillin-resistant CoPS (MR-CoPS) and vancomycin-resistant CoPS (VR-CoPS).

For the disc diffusion method, the inoculums of the microorganisms were adjusted to 0.5 of the McFarland scale and seeded onto plates containing Müeller–Hinton agar. The cefoxitin (CTX) (30 *μ*g), teicoplanin (TEC) (30 *μ*g), oxacillin (OXA) (1 *μ*g), and vancomycin (VAN) (5 *μ*g) disks (Laborclin®, Paraná, Brazil) were deposited, and the plates were incubated at 35 ± 2°C for 24 h for further analysis of the results by reading the inhibition halos using the CLSI cutoff points [14]. CoPS were considered resistant to CTX, TEC, and VAN when the inhibition halos were below 24, 16, and 17 mm, respectively.

The microdilution test was performed to determine the minimum inhibitory concentration (MIC). Initially, Müeller–Hinton broth was deposited in all wells of the microdilution plates. OXA or VAN (Sigma-Aldrich®, Missouri, USA) was distributed through serial dilution, obtaining concentration ranged from 0.25 to 128 *μ*g/mL and 0.0625 to 32 *μ*g/mL, respectively. The bacterial inoculums were adjusted to 0.5 of the McFarland scale, diluted, and deposited on the plates at the concentration of 10^5^ UFC/mL. Finally, the plates were incubated at 35 ± 2°C for 24 h, and after incubation, the MIC was determined as the lowest concentration where there was no microbial growth. The susceptibility profile was determined through the CLSI cutoff points [14]. CoPS were considered resistant to OXA and VAN when the MICs were above 0.5 and 4 *μ*g/mL, respectively.

In the agar screening for OXA and VAN, initially, they prepared plates containing MHA, 4% NaCl, and 6 *μ*g/mL of OXA and plates containing brain heart infusion agar (BHIA) (HiMedia®, Mumbai, India) and 6 *μ*g/mL of VAN. Then, the inoculums of the microorganisms were adjusted to 0.5 of the McFarland scale and seeded in the plates. Subsequently, the plates were incubated at 35 ± 2°C for 24 h, and after incubation, the plates were observed against the light for observation of bacterial growth. Any growth after 24 hours of incubation was considered resistant to OXA and/or VAN [14].

#### 2.6.2. Antimicrobial Susceptibility Tests of *Enterobacteriaceae*

The identification of the resistance profile of *Enterobacteriaceae* was carried out according to the Clinical and Laboratory Standards Institute [14]. The tests used in this study were the disk diffusion method, approximation test, and Hodge test.

In the disk diffusion method, the inoculums of the microorganisms were adjusted to 0.5 of the McFarland scale and seeded onto plates containing MHA. The ceftazidime (CAZ) (30 *μ*g), CTX (30 *μ*g), cefpodoxime (CPD) (10 *μ*g), aztreonam (ATM) (30 *μ*g), imipenem (IMP) (10 *μ*g), and meropenem (MEM) (10 *μ*g) disks (Laborclin®, Paraná, Brazil) were deposited, and the plates were incubated at 35 ± 2°C for 24 h for further analysis of the results by reading the inhibition halos using the CLSI cutoff points [14]. The approximation and Hodge tests were performed with the resistant strains in the disk diffusion method.

The approximation test was performed to determine the ESBL. Initially, the inoculums of the microorganismswere adjusted to 0.5 of the McFarland scale and seeded onto plates containing MHA. Thereafter, the amoxicillin/clavulanic acid (AMC) (10 *μ*g) disk (Laborclin®, Paraná, Brazil) was added in the center of the plates, and ATM and CAZ disks have been added with a distance of 20 to 25 mm from the AMC, on the sides of the plate. The plates were incubated at 35 ± 2°C for 24 h, and it was analyzed if there was an increase in the diameter of the inhibition halo or the appearance of the phantom zone, distortion of the halo around the *β*-lactam disk [14].

The Hodge test was performed to determine the KPC-producing bacteria. Initially, *Escherichia coli* ATCC 25922 was adjusted to 0.5 of the McFarland scale and seeded onto plates containing MHA. In the center of the plates was added a MEM disk. Subsequently, with the help of a handle, samples tests were striated near the *β*-lactam disk. The plates were incubated at 35 ± 2°C for 24  h, and it was analyzed for the presence of distortion in the inhibition halo, which is indicative of carbapenemase production [14].

#### 2.6.3. Antifungal Susceptibility Testing for Yeasts

Reference microdilution trays, containing serial drug dilutions, were prepared by following the CLSI M27-A3 guidelines [15]. Initially, the RPMI 1640 medium (Sigma-Aldrich®, Missouri, USA) was buffered to pH 7.0 with 0.165 M of morpholinopropanesulphonic acid (MOPS) (Sigma-Aldrich®, Missouri, USA).

In order to obtain a yeast inoculum containing 1–5 × 10^6^ CFU/mL, each strain was cultured on a tube containing 20 mL of Sabouraud dextrose agar (SDA) (HiMedia®, Mumbai, India) plus yeast extract at 35°C for 24 h for *Candida*, *Rhodotorula*, and *Trichosporon* isolates and 48 h for *Cryptococcus* isolates. After this time, yeast suspensions were prepared in sterile physiological solution (0.85%) and maintained at 28 ± 2°C and then were adjusted to 90% transmittance at 530 nm. Two serial dilutions from 1 : 100 and 1 : 20 sequentially were made to obtain a final inoculum containing 0.5 × 10^3^ to 2.5 × 10^3^ CFU/mL. The reference strain *Candida parapsilosis* ATCC 22019 was used. The standard antifungal drugs used were fluconazole (Pfizer Inc., New York, USA) and amphotericin B (Bristol-Myers Squibb, Princeton, USA). These drugs were dissolved in deionized water and dimethylsulfoxide, respectively, and tested in concentration ranged from 0.0312 to 64 *μ*g/mL.

The microplates were incubated at 35°C in a non-CO_2_ incubator and were visually evaluated after 48 h. The MIC corresponded to the lowest drug dilution that showed growth inhibition at least 50% when compared to untreated yeasts for fluconazole and that completely growth inhibited when compared to untreated inoculum for amphotericin B. The tests were performed in duplicate.

### 2.7. Evaluation of Biofilm Production in Bacteria and Yeasts

#### 2.7.1. Congo Red Agar Test

The qualitative determination of biofilm production by bacterial and fungal isolates was performed according to the red Congo agar method [16, 17]. Bacterial and fungal isolates were adjusted to 0.5 of the McFarland scale (10^8^ CFU/mL) in BHIB, incubated at 35 ± 2°C for 24 h, and seeded on plates containing red Congo agar medium (HiMedia®, Mumbai, India). Subsequently, they were incubated in an aerobic environment at 35 ± 2°C for 48 h. After this period, the colonies that presented blackish coloration, with dry or rough consistency, were considered as biofilm producers. Red colonies with mucosal consistency were considered as non-biofilm producers.

#### 2.7.2. Crystal Violet Staining Method

The quantification of biofilm production by bacteria and yeasts was performed by the crystal violet staining method reported by Stepanovié et al. [18] and Marcos-Zambrano et al. [19], respectively. For bacterial biofilm production, the bacteria were adjusted in tryptone soybean broth (TSB) (HiMedia®, Mumbai, India) supplemented with 1% glucose in the 0.5 McFarland scale (10^8^ CFU/mL) and distributed in microdilution plates. For fungal biofilm production, the yeasts were adjusted in RPMI 1640 medium on the 0.5 McFarland scale (10^6^ CFU/mL) and distributed in microdilution plates. The microplates of bacteria and fungi were incubated at 35 ± 2°C for 48 h. After incubation, the supernatant was removed, and the wells were washed with phosphate buffer pH 7.4. Then, 200 *μ*L of 99% methanol was added for 15 minutes, and then the wells were emptied. Subsequently, the crystal violet (Sigma-Aldrich®, Missouri, USA) was added to the plates, which were incubated at 37°C for 30 min. Thereafter, the contents of the wells were removed, and the wells were washed with phosphate buffer and glacial acetic acid was added. Finally, the optical density (OD) was measured by spectrophotometry at 570 nm (Multiskan microplate photometer FC, Thermo scientific, Madrid, Spain).

Wells containing only culture medium were used as controls. The strains were classified into four categories based on the OD values of the biofilms compared to ODc (optical density of control). The strains were classified as nonadherent if OD ≤ ODc; poor yield if ODc < OD ≤ 2 × ODc; moderate production if 2 × ODc < OD ≤ 4 × ODc; or strong production if 4 × ODc < OD [14].

## 3. Results

### 3.1. Identification and Characterization of Antimicrobial Resistance Profile and Biofilm Production of CoPS

CoPS was identified in eight (67%) of the 12 birds analyzed, represented by all specimens of *R. magnirostris* and two specimens of *C. plancus* (Table 1). Regarding the antimicrobial resistance profiles analyzed, the concomitant profile MR- and VR-CoPS were identified in 50% of the samples; only the profile MR-CoPS was present in 12.5%, and 37.5% of the CoPS isolated from the oropharynx of the birds was antibiotic-sensitive. Considering the presence of these bacteria in the *R. magnirostris* specimens, it was identified that an incidence of 50% of antibiotic-sensitive CoPS, 33% of MR, and VR-CoPS concurrently and 17% of only MR-CoPS. While in the *C. plancus*, VR-CoPS strains were present in 100% of the samples (Table 1).

**Figure 1:**
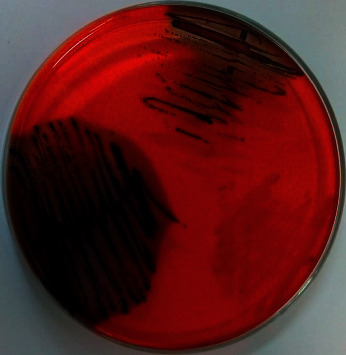
Biofilm production by coagulase-positive staphylococci isolated from the oropharynx of *Rupornis magnirostris* and *Caracara plancus* by the red Congo agar method after 24 hours at 35°C.

Regarding the biofilm production, the results of the qualitative method using the Congo red agar were faithful to the results of the quantitative method performed by the violet crystal staining. Among the 8 CoPS isolates, 3 were biofilm producers in the qualitative method (37.5%) (Figure 1). From the data of the quantitative method, it was possible to observe that of these 3 isolates that were biofilm producers, 2 isolates were weak biofilm producers (66.7%) and one isolate was strong a biofilm producer (33.3%). In the *R. magnirostris* specimens, 67% of the samples was considered as nonbiofilm producers, 17% as strong producers, and 16% as weak producers. On the other hand, in the *C. plancus* specimens, 50% of the samples was weak biofilm producers and the other 50% was strong producers (Table 1).

### 3.2. Identification and Characterization of Antimicrobial Resistance Profile and Biofilm Production of *Enterobacteriaceae*

Nine animals had enterobacteria (75%), including all specimens of *R. magnirostris* and three birds of the *C. plancus*. Twenty-seven species of enterobacteria were isolated, and in all the animals, the co-occurrence of two to four different species was identified. The most frequently isolated pathogen was *Escherichia coli* (25%), isolated from 6 animals, followed by *Salmonella* spp. (25%), *Klebsiella oxytoca* (15%), and *Proteus mirabilis* (15%) identified in four animals. Besides *Shigella* spp. (10%) and *Klebsiella pneumoniae* (10%) isolated from three animals (Table 2).

Related to the antimicrobial resistance profile, of the 27 enterobacteria isolated from the animals, 14 strains were antibiotic‐sensitive enterobacteria (51.9%), 8 strains were extended-spectrum beta-lactamase-producing *Enterobacteriaceae* (ESBL) (29.6%), 3 isolates were third-generation cephalosporins-resistant *Salmonella* spp. (11.1%), and 2 isolates were KPC-producing bacteria (7.4%). The results for each species showed the presence of 22.2%, 11.1%, and 11.1% of the ESBL, third-generation cephalosporins-resistant *Salmonella* spp., and KPC, respectively, in the 18 samples from *R. magnirostris*, while in the 9 bacteria isolated from *C*. *plancus*, 44.4% was ESBL and 11.1% was third-generation cephalosporins-resistant *Salmonella* spp. (Table 2).

Regard to the biofilm production, 16 from the 27 isolates of enterobacteria were biofilm producers in the qualitative method (59%). It was possible to observe that of these 16 biofilm-producing isolates, 4 isolates were weak producers (25%) and 12 isolates were strong biofilm producers (75%). Taking into account the analyzed species, it was identified that 37% of the samples from *R*. *magnirostris* specimens was not biofilm producers, 5% were weak producer, and 58% were moderate biofilm producers. While in the *C. plancus* specimens, 50% of the samples was identified as nonbiofilm producers, 37% was weak producer, and 13% was moderate biofilm producers (Table 2).

### 3.3. Identification and Characterization of Antimicrobial Resistance Profile and Biofilm Production of Yeasts

Yeasts were identified in 7 of the 12 animals (58.3%), being represented by 4 animals of the *R. magnirostris* specimens and 3 of the *C*. *plancus* specimens, totalizing 10 yeast isolates. Among these 10 yeasts, 2 were identified as *Candida metapsilosis* (20%), 2 were *Rhodotorula mucilaginosa* (20%), 2 were *Trichosporon coremiiform* (20%), 1 was *Rhodotorula glutinis* (10%), 1 was *Cryptococcus* sp. (10%), 1 was *Candida* sp. (10%), and 1 was *Candida ciferri* (10%) (Table 3).

Regarding the susceptibility profile of yeast isolates, it was possible to observe that *Rhodotorula mucilaginosa* isolates showed resistance to fluconazole and susceptibility to amphotericin B. However, the isolates of *Trichosporon coremiiform* showed resistance to amphotericin B and susceptibility to fluconazole. *Candida metapsilosis*, *Candida ciferri*, and *Rhodotorula glutinis* showed dose-dependent susceptibility to fluconazole but were susceptible to amphotericin B (Table 3).

As for the biofilm production, of the 10 yeast isolates, 7 were biofilm producers (70%), and all were classified as weak biofilm producers (100%). The *R. magnirostris* specimens had 83% of biofilm former and 17% of nonformer specimens, whereas *C. plancus* presented 50% of weak biofilm producer samples and the other 50% had no biofilm producer ability (Table 3).

## 4. Discussion

We highlight that the study of dissemination of biofilm-forming microorganisms with an antimicrobial resistance profile is of interest to human and animal health [9]. In this context, the identification of these pathogens in animals, such as birds, is very relevant.

Recent studies have also identified *Staphylococcus* spp. in birds. Dipineto et al. [20] identified the presence of 87.7% of *S. aureus* in bacterial isolates from birds of prey. Concerning antimicrobial resistant *S. aureus* samples, other studies have already isolated these bacteria in several anatomical sites of wild birds. Loncaric et al. [5] also identified isolates of *Staphylococcus aureus* with resistance to methicillin in faecal samples from a migratory population of *Corvus frugilegus* and suggest that these genotypes originated from humans and cattle, but were disseminated affecting wild organisms.

The reports of VR-CoPS identification in birds are scarce, since vancomycin is used in a limited way in treatments of some infections. However, isolates of VR-CoPS have been previously reported in birds at Brazil, being the first report of resistance to this antimicrobial carried out by Martins et al. [21]. These authors also describe the presence of both *van*A and *van*B genes in all vancomycin-resistant isolates. Therefore, it is possible to state that there is already an unusual description of *Staphylococcus* spp. with resistance to vancomycin in birds at Brazil. In our study, 50% of CoPS isolates were VR-CoPS. Resistance to vancomycin also occurs in other species of the genus *Staphylococcus*, as reported in the study developed by Ishihara et al. [22] that identified biofilm-forming *Staphylococcus succinus* with vancomycin resistance genes isolated from saliva of wild songbirds.

Data related to avian biofilm-forming VR-CoPS are also rare; among the few studies, we emphasize the study of Nemati et al. [23] that identified genes associated with biofilm production in *S. aureus* isolates from the nostrils, skin, cloaca of healthy animals, and head injuries of poultry farms.

Enterobacteria were a group of bacteria that can be part of the intestinal microbiota of animals; nevertheless, this microbiota can develop the ability to cause gastrointestinal infections and damages the enteric area of humans and animals [20].

Several studies have reported the presence of enterobacteria, such as *E. coli*, *Salmonella* spp., *Klebsiella* spp., *Proteus* spp., and *Shigella* spp., in anatomical sites of birds of prey, such as cloacal samples of gavião-carijó, cloacal and pharyngeal samples of the griffon vulture (*Gyps fulvus*), cloaca samples of hawk-carijó, and faecal samples from resident and migratory population of rooks (*Corvus frugilegus*), in several countries around the world [5, 8, 18, 20, 24–26].

*Enterobacteriaceae* family with antimicrobial resistance profiles is considered a public health threat due to their zoonotic potential [5]. Micro-organisms of the ESBL-producing *Enterobacteriaceae* family found in free-living animals have been reported, mostly in birds. Although several bacteria from the *Enterobacteriaceae* group presented this resistance profile, ESLB was more frequently identified in *E. coli* followed by *K. pneumoniae* [27]. Loncaric et al. [5] identified ESBL *Enterobacteriaceae* isolates in faecal samples from resident and migratory population of rooks (*Corvus frugilegus*) in Austria.

*Klebsiella pneumoniae* carbapenemase (KPC) are the most common class A carbapenemases and are mostly encoded by *K. pneumoniae* enzymes. In humans, infections associated with the KPC profile tend to present systemic conditions, causing serious clinical conditions that are difficult to treat due to limited antibiotic options [28]. Regarding the identification of KPC-producing bacteria in animals, the data in the literature are scarce and restricted to domestic animals, such as the study of Stolle et al. [29] that identified this profile in dogs.

Some species of the family *Enterobacteriaceae* are known to produce biofilm, including *E. coli* and *Salmonella* spp. In the study performed by Olson et al. [30], *E. coli* biofilms isolated from poultry, swine, and cattle were resistant to most of the antimicrobials analyzed. In the study performed by Nandanwar et al. [31], the results showed that the isolated pathogenic *E. coli* strains from birds and mammals were able to form biofilms, besides being resistant to the bactericidal activity of human and avian sera, highlighting the potential zoonotic risk.

Some studies indicate the presence of biofilm-producing *Enterobacteriaceae* in breeding birds and by-products. Chuah et al. [32] analyzed enteric strains of *Salmonella* species from samples of birds and environmental samples collected from wet-markets in northern Malaysia. In this study, all strains were biofilm-producing, and 69.3% of them were strong biofilm producers. Studies that analyze the presence of *Salmonella* spp. consider this biofilm as a source of constant contamination of poultry production systems, generating economic and public health impacts [33].

Several species of yeast are pathogenic to humans and animals, including free-living birds. Thus, the present study was linear with the previous study, which reported the presence of *Rhodotorula* spp., *Cryptococcus* spp., and *Trichosporon* spp. isolated in samples from birds, such as pigeons, passeriform feces samples in captivity, or samples of pappus lesions [34–37]. Representatives of the genus *Candida* cause candidiasis, a pathology that usually affects cutaneous and mucosal regions, as well as genital and gastrointestinal tract of animals and humans. In birds, there are reports of candidiasis mainly affecting the upper digestive system, with emphasis on the oropharynx and esophagus [38]. In a study with birds of prey, Samour and Naldo [38] identified candidiasis through clinical signs and positive cultures for *C*. *albicans*.

Mendes et al. [39] also shown the presence of some fungal strains in their analyzes in wild bird feces, reporting the presence of *Candida* spp., *Trichosporon asahii*, *Rhodotorula* sp., and *Cryptococcus laurentii*. Resistance to antimicrobial agents in yeasts isolated from birds has been described in the literature. Lord et al. [34] also identified resistance yeasts, such as *C. albicans*, *C. laurentii*, *T. pullulans*, *R. rubra*, and *R. glutinis*, in faecal samples from wild birds, and Costa et al. [35] identified strains of resistant *Cryptococcus* spp. in faecal samples of pigeons.

The most relevant results observed in this study and a matter of serious concern was the concomitant presence of micro-organisms with resistance profile and biofilm producers, such as 1MR- and VR-CoPS strains are strong producers of biofilm, 2 ESBL, 3 third-generation cephalosporins-resistant *Salmonella* spp., and 1 KPC strains are moderate producers of biofilm, 1 ESBL strain is a weak producer of biofilm, 1 *Trichosporon coremiiforme* strain is resistant to amphotericin B and a weak producer of biofilm, and 2 *Rhodotorula mucilaginosa* strains are resistant to fluconazole and are weak producers of biofilm.

Furthermore, the animals surveyed were in a Wild Animals Triage Center of the Environment agency of the Pernambuco state responsible for the care of local fauna. Thus, as in other studies, these animals come to be screened for complaints, accidents, and criminal capture and may be subsequently screened, kept in captivity for treatment and rehabilitation and for subsequent environmental reintegration, and some may remain in this center because they are not physically able to return to the natural environment [40, 41].

Since the collection of samples from the animals was performed before they were submitted to antibiotic therapy, we believe that the isolated micro-organisms actually emerged to microbiota of these animals and bring a close panorama of what contact they had in the natural environment where they lived before their capture. The space in which the animals were found was shared, including healthy animals, ready for environmental reintegration, sick and injured animals, as well as mutilated animals that remain in the screening center, which generates a potential for contamination between them. We emphasize that *Rupornis magnirostris* and *Caracara plancus* are well adapted to urban environments, especially *Caracara plancus* [42]. So these animals may have been contaminated by the environment and may have transmitted these pathogens among other animals that also returned to the natural environment, indicating that these bird of prey can be potential carriers and disseminators of resistant microorganismss and/or biofilm producers to other environments, other animals, and in humans, causing infections that are considered public health problem because all these bacteria are human pathogens that play an important role in drug resistance dissemination and potentialize several serious human infections, including septicemia and death [43–45]. In accordance with other studies, we also alert to the appropriate use of antibiotics in agriculture, livestock, veterinary medicine, and human medicine, avoiding the risk of the spread of zoonotic infections caused by drug-resistant microorganisms[46, 47].

A very probable hypothesis that explains the presence of resistant and/or biofilm-producing microorganismsin the birds of the present study is the fact that, in Brazil, laws regulate the production of medicines by pharmaceutical companies, but there is a lack of effective surveillance regarding the disposal of medicines. Thus, these medicines can reach the environment due to their incorrect disposal by the final consumer, who often discards these drugs directly in the garbage, or due to the untreated industrial waste [48]. Therefore, it is important to encourage studies to discover cross-transmission from animal to human and vice versa, and the role of the environment in this process.

## 5. Conclusion

Bearing in mind all these findings, the results of this study are relevant and significant as a preliminary study to emphasize the importance to fill the existing gap in veterinary medicine with regard to the identification of resistant microorganisms and biofilm producers in birds of prey species. *Rupornis magnirostris* and *Caracara plancus* have pathogenic micro-organisms of interest for human public health, such as CoPS, enterobacteria, and yeasts, in addition to species with resistance profile to several antimicrobials and/or with biofilm production capacity. Our findings highlight the potential of these birds as a reservoir of microorganisms, in addition to being able to indicate a possible dissemination of pathogens in the environmental, human, and animal health, due to the convergence between urban and wild habitats.

## Figures and Tables

**Table 1:** Resistance profile and biofilm production of coagulase-positive staphylococci isolated from oropharynx of *Rupornis magnirostris* and *Caracara plancus*.

Animals	Screening		Microdilution (MIC *μ*g/mL)		Disk diffusion (inhibition zone mm)			Resistance profile	Biofilm production	
	OXA	VAN	OXA	VAN	CTX	TEC	VAN		Congo red	Crystal violet
*Rupornis magnirostris* 1	−	−	1	0.5	28.2 ± 2.2	20.4 ± 2.2	24.6 ± 1.2	Antibiotic-sensitive CoPS	−	Nonproducer
*Rupornis magnirostris* 2	+	+	>128	>32	15.4 ± 1.9	0	0	MR- and VR-CoPS	+	Weak
*Rupornis magnirostris* 3	−	−	1	0.5	32.8 ± 1.3	21.9 ± 2.1	24.2 ± 0.4	Antibiotic-sensitive CoPS	−	Nonproducer
*Rupornis magnirostris* 4	+	−	128	0.5	16.2 ± 0.5	23.1 ± 1.2	23.0 ± 0.9	MR-CoPS	−	Nonproducer
*Rupornis magnirostris* 5	−	−	≤0.25	0.5	28.4 ± 1.0	21.5 ± 1.6	24.7 ± 1.4	Antibiotic-sensitive CoPS	−	Nonproducer
*Rupornis magnirostris* 6	+	+	>128	>32	14.7 ± 1.3	0	0	MR and VR-CoPS	+	Strong
*Caracara plancus* 1	+	+	>128	>32	13.0 ± 0.5	0	0	MR and VR-CoPS	+	Weak
*Caracara plancus* 2	+	+	>128	>32	13.9 ± 0.8	0	0	MR and VR-CoPS	−	Nonproducer

MIC: minimum inhibitory concentration; OXA: oxacillin; VAN: vancomycin; CTX: cefoxitin; TEC: teicoplanin; and CoPS: coagulase-positive staphylococci.

**Table 2:** Resistance profile and biofilm production of *Enterobacteriaceae* isolated from oropharynx of *Rupornis magnirostris* and *Caracara plancus.*

Animals	Bacteria identifications	Inhibition zone (mm)						Approximation test	Hodge test	Resistance profile	Biofilm production	
		CAZ	CTX	CPD	ATM	IMP	MEM				Congo red	Crystal violet
*Rupornis magnirostris* 1	*Salmonella* spp.	S	S	S	S	S	S	NP	NP	NR	+	Moderate
	*Klebsiella oxytoca*	S	S	S	S	S	S	NP	NP	NR	+	Weak
	*Shigella* spp.	S	S	S	S	S	S	NP	NP	NR	−	Nonproducer
	*Proteus mirabilis*	R	R	R	R	NP	S	+	−	ESBL	−	Nonproducer

*Rupornis magnirostris* 2	*Klebsiella pneumoniae*	S	S	S	S	S	S	NP	NP	NR	+	Moderate
	*Escherichia coli*	R	R	R	R	S	S	+	−	ESBL	−	Nonproducer
	*Salmonella* spp.	R	R	R	R	S	S	+	−	Third-generation cephalosporins	+	Moderate

*Rupornis magnirostris* 3	*Salmonella* spp.	S	S	S	S	S	S	NP	NP	NR	+	Moderate
	*Klebsiella oxytoca*	R	R	R	R	R	R	−	+	KPC	+	Moderate
	*Escherichia coli*	R	R	R	R	R	R	−	+	KPC	−	Nonproducer

*Rupornis magnirostris* 4	*Escherichia coli*	S	S	S	S	S	S	NP	NP	NR	−	Nonproducer
	*Salmonella* spp.	S	S	S	S	S	S	NP	NP	NR	+	Moderate
	*Proteus mirabilis*	S	S	S	S	NP	S	NP	NP	NR	−	Nonproducer

*Rupornis magnirostris* 5	*Salmonella* spp.	S	S	S	S	S	S	NP	NP	NR	+	Moderate
	*Klebsiella pneumoniae*	S	S	S	S	S	S	NP	NP	NR	+	Moderate
	*Proteus mirabilis*	R	R	R	R	NP	S	+	−	ESBL	−	Nonproducer

*Rupornis magnirostris* 6	*Shigella* spp.	R	R	R	R	S	S	+	−	ESBL	+	Moderate
	*Salmonella* spp.	R	R	R	R	S	S	+	−	Third-generation cephalosporins	+	Moderate
	*Escherichia coli*	R	R	R	R	S	S	+	−	ESBL	+	Moderate

*Caracara plancus* 2	*Salmonella* spp.	R	R	R	R	S	S	+	−	Third-generation cephalosporins	+	Moderate
	*Escherichia coli*	S	S	S	S	S	S	NP	NP	NR	−	Nonproducer

*Caracara plancus* 4	*Klebsiella oxytoca*	R	R	R	R	S	S	+	−	ESBL	−	Nonproducer
	*Klebsiella pneumoniae*	R	R	R	R	S	S	+	−	ESBL	+	Weak
	*Proteus mirabilis*	S	S	S	S	NP	S	NP	NP	NR	+	Weak

*Caracara plancus* 5	*Shigella* spp.	S	S	S	S	S	S	NP	NP	NR	−	Nonproducer
	*Escherichia coli*	R	R	R	R	S	S	+	−	ESBL	−	Nonproducer
	*Klebsiella oxytoca*	S	S	S	S	S	S	NP	NP	NR	+	Weak

S: sensible; R: resistant; NR: no resistance; NP: not performed; CAZ: ceftazidime; CTX: cefoxitin; CPD: cefpodoxime; ATM: aztreonam; IMP: imipenem; MEM: meropenem; ESBL: extended-spectrum *β*-lactamase-(ESBL-) producing *Enterobacteriaceae*; and KPC: *Klebsiella pneumoniae* carbapenemase.

**Table 3:** Resistance profile and biofilm production of yeasts isolated from oropharynx of *Rupornis magnirostris* and *Caracara plancus*.

Animals	Identification of species	MIC_AmB_(*μ*g/mL)	Susceptibility	MIC_FLU_(*μ*g/mL)	Susceptibility	Biofilm production	
						Congo red	Crystal violet
*Rupornis magnirostris* 1	*Candida metapsilosis*	0.25	S	0.50	S	+	Weak
*Rupornis magnirostris* 2	*Rhodotorula mucilaginosa*	1	S	64	R	+	Weak
*Rupornis magnirostris* 3	*Candida metapsilosis*	0.125	S	1	S	−	Nonproducer
	*Candida* sp.	0.06	S	2	S	+	Weak
	*Cryptococcus* sp.	0.50	S	4	S	+	Weak
*Rupornis magnirostris* 5	*Rhodotorula mucilaginosa*	1	S	64	R	+	Weak
*Caracara plancus* 1	*Candida ciferri*	1	S	16	SDD	+	Weak
*Caracara plancus* 2	*Rhodotorula glutinis*	0.50	S	32	SDD	−	Nonproducer
	*Trichosporon coremiiforme*	4	R	0.0125	S	−	Nonproducer
*Caracara plancus* 3	*Trichosporon coremiiforme*	4	R	0.06	S	+	Weak

MIC_AmB_: minimum inhibitory concentration of amphotericin B; MIC_FLU_: minimum inhibitory concentration of fluconazole; S: susceptible; R: resistant; SDD: susceptible dose-dependent; +: producer of biofilm; and −: nonproducer of biofilm.

## Data Availability

The data used to support this study are included within the article and will be made available upon request.
